# Liberal Versus Restrictive Transfusion in Acute Brain Injury: A Systematic Review and Meta-Analysis

**DOI:** 10.7759/cureus.83847

**Published:** 2025-05-10

**Authors:** Joseph Yvan Bena Nnang, Ticha Brandon Tita Tembi, Samuel G Fodop, Joel Gabin Konlack Mekontso, Ignatius N Esene

**Affiliations:** 1 General Medicine, Faculty of Medicine and Biomedical Sciences, Université de Yaoundé I, Yaounde, CMR; 2 General Medicine, Hubert Department of Global Health, Rollins School of Public Health, Emroy University, Atlanta, USA; 3 Medicine, Catholic University of Cameroon, Bamenda, CMR; 4 Internal Medicine, New York City Health + Hospitals, South Brooklyn Health, New York City, USA; 5 Neurosurgery, Faculty of Health Sciences, University of Bamenda, Bambili, CMR

**Keywords:** acute brain injury, liberal transfusion, restrictive transfusion, subarachnoid hemorrhage, traumatic brain injury

## Abstract

Oxygen is critical for neurological function and survival, particularly in acute brain injury. Although transfusion at higher thresholds theoretically provides improved oxygen delivery to neurons, there is an associated risk of allogeneic reactions and increased utilization of limited blood resources. Conversely, although a lower threshold conserves resources, it may increase the risk of neuronal oxygen deprivation. The optimal transfusion strategy for patients with acute brain injury remains unclear. This systematic review and meta-analysis aimed to compare the efficacy and safety of liberal (hemoglobin threshold ≤10 g/dL) versus restrictive (≤8 g/dL) transfusion strategies in patients with acute brain injury (traumatic brain injury (TBI), subarachnoid hemorrhage, or intracranial hemorrhage), synthesizing evidence from randomized controlled trials (RCTs).

We searched the PubMed, Excerpta Medica database (Embase), and the Cochrane Central Register of Controlled Trials (CENTRAL) databases to identify RCTs comparing restrictive and liberal transfusion strategies in patients with acute brain injury. Eligible trials reported outcomes including (1) unfavorable neurological outcomes defined as a score ≤5 on the Glasgow Outcome Scale Extended (GOSE) at six months, (2) mortality, (3) acute respiratory distress syndrome (ARDS), and (4) infections. Statistical heterogeneity was assessed using I² statistics. To account for statistical heterogeneity, a random-effects model was used to analyze all outcomes.

We included six RCTs comprising 2,645 patients, of whom 1,303 (49.2%) were randomized to a liberal transfusion strategy. A reduction in unfavorable neurological endpoints (55.7% vs. 61.4%; risk ratios (RR) 0.92; 95% CI 0.84-1.01) was observed in the liberal group, although this difference was not statistically significant. In sensitivity analyses of unfavorable neurologic outcomes, statistical significance was achieved by removing a single paper (54.7% vs. 61.6%; RR 0.89; 95% CI 0.84-0.95) or by employing a fixed-effects model (RR 0.91; 95% CI 0.85-0.97).

This meta-analysis suggests that there is no substantial difference in outcomes between a liberal and a restrictive transfusion strategy in patients with acute brain injury. While our primary analysis showed no statistically significant difference between strategies, sensitivity analyses suggested a potential benefit of liberal transfusion in reducing unfavorable neurologic outcomes. However, given the non-significant primary results and the importance of blood conservation, a restrictive strategy may be reasonable until further evidence emerges.

## Introduction and background

Globally, an estimated 27 to 69 million people experience a traumatic brain injury (TBI) each year, highlighting the significant worldwide burden of acute brain injuries [1,2].

A significant proportion of patients with acute brain injuries experience secondary insults, with studies reporting rates as high as 90% of patients experiencing at least one secondary insult. These factors are strongly associated with poor clinical outcomes and increased mortality rates [3-6]. 

In patients with acute brain injury, anemia can be a serious comorbidity, leading to secondary brain injury by reducing oxygen delivery to the vulnerable peri-lesional tissue, exacerbating secondary injury through hypoxic-ischemic mechanisms, and is associated with worse endpoints [7, 8]. Transfusion at higher thresholds (liberal) theoretically provides better oxygen delivery to neurons; however, there is a risk for transfusion reactions and great utilization of limited blood resources. Alternatively, a lower threshold (restrictive) would spare blood resources but potentially bear the risk of depriving neurons of oxygen when they need it the most. The optimal transfusion strategy for patients with acute brain injury remains uncertain [9].

Previous meta-analyses conducted on this topic were unable to detect significant differences between groups and focused essentially on patients with TBI. Earlier meta-analyses [10-12] were unable to detect significant differences between strategies, likely due to heterogeneity in study designs, inclusion of observational data with inherent confounding, and limited statistical power. Moreover, the lack of high-quality randomized data limits their ability to draw substantial conclusions due to the high risk of bias from observational studies [10-12].

With blood shortages affecting countries worldwide and growing recognition of transfusion risks, determining the optimal strategy has immediate implications for both patient outcomes and healthcare resource utilization. Our study addresses these limitations by (1) including only randomized controlled trials (RCTs) to minimize bias, (2) encompassing all major types of acute brain injury, and (3) incorporating recent large trials not included in prior analyses. While high-quality observational studies can provide valuable insights, they are prone to confounding by indication, particularly for transfusion decisions, which are often influenced by clinical factors associated with worse outcomes. We restricted our analysis to RCTs because they represent the highest level of evidence for evaluating therapeutic interventions.

## Review

Methods

This systematic review and meta-analysis was conducted following the guidelines of the Cochrane Collaboration Handbook of Systematic Review of Interventions and the Preferred Reporting Items for Systematic Review and Meta-analysis (PRISMA) statement [13,14].

Eligibility criteria

Inclusion in this meta-analysis was restricted to studies that met the following eligibility criteria: (1) RCTs; (2) comparing liberal transfusion (triggered by a hemoglobin level of ≤10 g/dL) versus restrictive transfusion (triggered by a hemoglobin level of ≤8 g/dL) strategies; and (3) enrolling patients with acute brain injuries. Additionally, studies were included only if they reported any clinical endpoints of interest, most notably unfavorable neurological outcome (defined as a score ≤5 on the Glasgow Outcome Scale Extended (GOSE) at six months).

In this study, acute brain injury was defined to include three specific types of brain injuries: (1) TBI, (2) aneurysmal subarachnoid hemorrhage, and (3) intracranial hemorrhage. Patients with any of these three types of brain injuries were eligible for inclusion in the study, provided they met other criteria.

We excluded studies with (1) an observational study design, (2) no control group, (3) platelet or plasma transfusions, (4) overlapping patient populations, (5) no endpoints of interest, and (6) non-English literature. There was no restriction on studies based on publication date.

While our protocol initially specified a restrictive threshold of ≤7 g/dL, we expanded this to ≤8 g/dL during study screening to include a recent large trial that used ≤8 g/dL as its restrictive threshold. This modification was made to enhance the clinical applicability of our findings while maintaining the fundamental contrast between restrictive (≤8 g/dL) and liberal (≤10 g/dL) strategies. All analyses were subsequently performed using this updated definition.

Search strategy and data extraction

We systematically searched PubMed, the Excerpta Medica database (Embase), and the Cochrane Central Register of Controlled Trials (CENTRAL) from inception to December 2024 with the search terms ‘traumatic brain injury’, ‘acute brain injury, ’ ‘head trauma, ’ ‘brain injuries, ’ 'subarachnoid hemorrhage, ’ ‘liberal, ’ ‘higher, ’ ‘blood transfusion, ’ ‘hemoglobin, ’ ‘blood products, ’ and ’packed red blood cells.’ The exact search strategy and Boolean terms are available in Appendix A.

The references of all included studies, previous systematic reviews, and meta-analyses were also manually searched for additional studies. Two authors (JYBN and TBTT) independently extracted the data using predefined search criteria and quality assessments. The prospective meta-analysis protocol was registered on the International Prospective Register of Systematic Reviews (PROSPERO) on November 8, 2024, under the protocol CRD42024607304.

Endpoints and sensitivity analyses

The endpoints included unfavorable neurological endpoints, all-cause mortality, intensive care unit (ICU) mortality, venous thromboembolism (VTE), infections, acute respiratory distress syndrome (ARDS), and length of hospital stay. An unfavorable neurological outcome was defined as a score ≤5 on the GOSE at six months.

Quality assessment

We evaluated the risk of bias in all studies using version 2 of the Cochrane Risk of Bias Assessment (RoB 2) tool [15]. Two independent authors (JYBN and TBTT) independently assessed the risk of bias. Disagreements were resolved through consensus after discussion of the reasons for the discrepancy. Publication bias was investigated using a funnel plot analysis of the point estimates to study weights.

Statistical analyses

Risk ratios (RR) with 95% confidence intervals were used to compare the treatment effects for categorical endpoints. Continuous endpoints were compared using mean differences. We assessed statistical heterogeneity using I² statistics and the Cochran's Q test; p-values < 0.10 and I² > 25% were considered significant for statistical heterogeneity. We used the random effects model given concern for differences in intervention effects across trials. 

We used Review Manager 8.13.0 (Cochrane Center, The Cochrane Collaboration, Denmark) for statistical analysis [16]. For continuous endpoints analysis, medians and interquartile ranges were used to estimate means and standard deviations, respectively, when no significant evidence of skewness was found [17-20]. Post-hoc power calculations were performed for all outcomes using the two-proportion test in R (pwr.2p2n.test), with alpha = 0.05 and the observed event rates.

Sensitivity analysis

We also performed two sensitivity analyses: (1) removing each study from the endpoint assessment and (2) analyzing endpoints using the fixed-effects model. 

We performed a leave-one-out sensitivity analysis to ensure that the results were not dependent on a single study and analyzed all endpoints using the fixed-effects model to ensure that the results did not depend on the choice of the analysis model. Studies with protocol deviations or confounding co-interventions (e.g., erythropoietin) were flagged for subgroup exclusion if they disproportionately affected results.

Risk of bias assessment

The methodological quality of included RCTs was evaluated using ROB 2 [15]. This tool assesses bias across five domains: (1) randomization process, (2) deviations from intended interventions, (3) missing outcome data, (4) outcome measurement, and (5) selective reporting. For each domain, studies were rated as "low risk," "some concerns," or "high risk" based on predefined signaling questions. Two independent authors (JYBN and TBTT) conducted the assessments, with disagreements resolved through consensus. Overall risk of bias for each study was determined by the highest level of bias identified across domains.

Certainty of evidence assessment

The Grading of Recommendations, Assessment, Development, and Evaluation (GRADE) framework was used to assess the certainty of evidence for each outcome in our meta-analysis. GRADE evaluates evidence based on five key domains with the potential to downgrade or upgrade the certainty rating: risk of bias, inconsistency, indirectness, imprecision, and publication bias. We utilized the GRADEPro GDT software (McMaster University and Evidence Prime, 2025. Available from gradepro.org). Overall, all results were of moderate certainty [21-24]. The GRADE Working Group grades of evidence are as follows: High certainty: We are confident that the true effect lies close to that of the estimate of the effect; Moderate certainty: We are moderately confident in the effect estimate; the true effect is likely to be close to the estimate of the effect, but there is a possibility that it is substantially different; Low certainty: Our confidence in the effect estimate is limited; the true effect may be substantially different from the estimate of the effect. Very low certainty: We have very little confidence in the effect estimate; the true effect is likely to be substantially different from the estimate of effect.

Results

Study Selection and Baseline Characteristics

**Figure 1:**
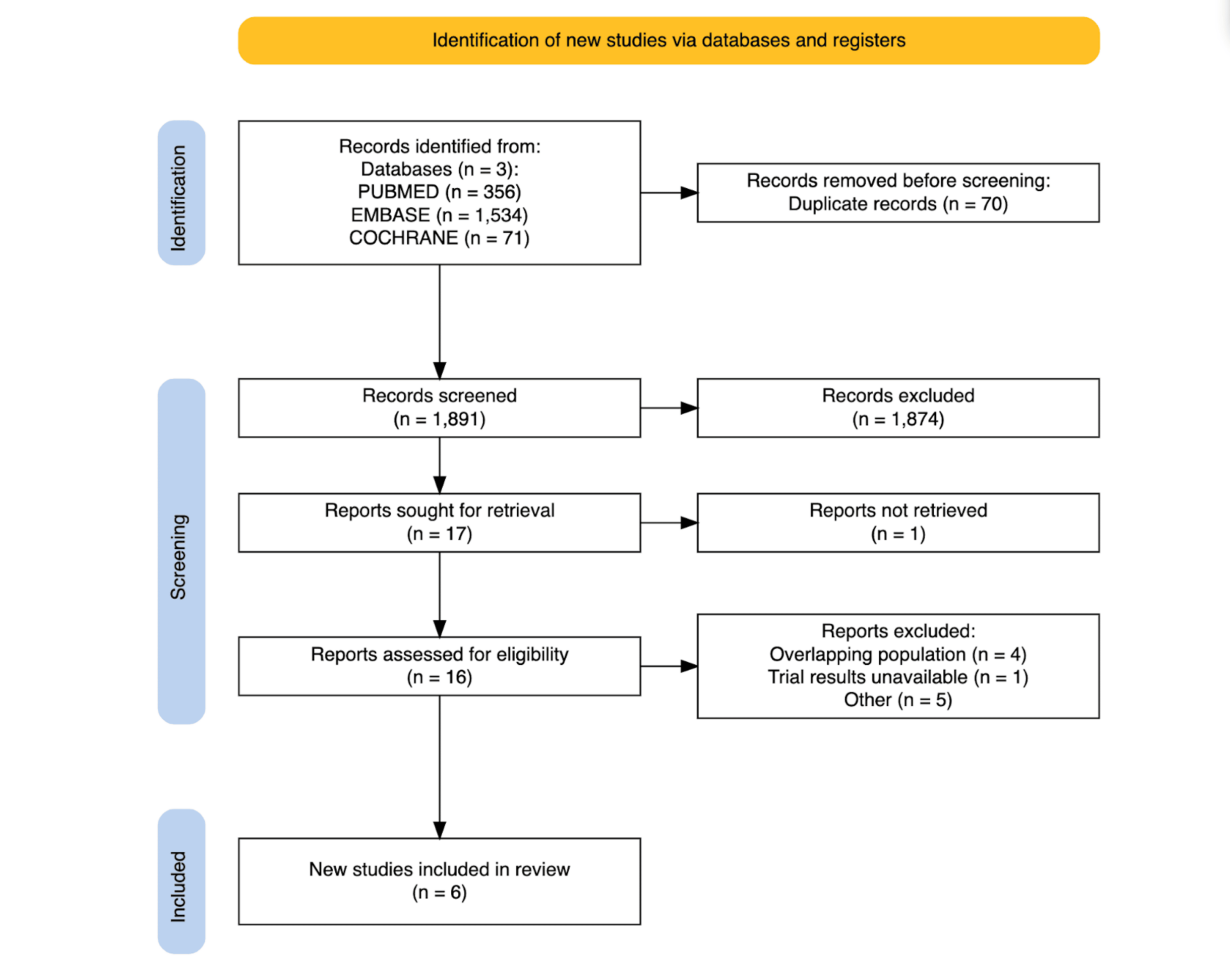
PRISMA flow diagram of study screening and selection PRISMA: PRISMA: Preferred Reporting Items for Systematic Reviews and Meta-Analyses; Embase: Excerpta Medica database

The initial search yielded 1,961 results. After removing duplicates and ineligible studies based on title/abstract review, 17 studies remained and were fully reviewed based on the inclusion criteria. Six studies were included [25-30], comprising 2,645 patients from RCTs (Figure 1) [31]. Studies that appeared to meet the inclusion criteria but were excluded are cited in Appendix B.

A total of 1,303 (49.3%) patients were randomized to the liberal transfusion strategy group and 1,342 (50.7%) to the restrictive strategy group. The study’s characteristics are presented in Table 1. The mean age of the participants across studies ranged from 29 to 51.5 years. Most studies included patients with TBI, except two, which reported 190 (22.4%) [25] and 742 (100%) [30] of patients with subarachnoid hemorrhage. Baseline hemoglobin levels across studies ranged from 8.1 to 14.5 g/dL. Overall, 1310 (49.5%) patients were reported to have reactive pupils. No significant differences were observed in the baseline characteristics of the studies (Table 1).

**Table 1:** Baseline characteristics of included studies ^a^mean or median; ^b^Erythropoietin was concurrently administered to patients in this study; ^c^Specific denominators are provided for variables with missing data. GCS: Glasgow Coma Scale; Hb: hemoglobin; NA: not available; TBI: traumatic brain injury

Study details		Patients	Age (years)^a^	Female, n (%)	TBI, n (%)	GCS score^a^	Pupillary reactivity			Base glucose^a^ mg/dL	Base Hb^a^ g/dL	Liberal Hb threshold	Restrictive Hb threshold
							Both	One	None				
English et al., 2024 [30]		742	60	299 (81.7)	NA	NA	NA	NA	NA	NA	9.3	10 g/dLor less	8 g/dLor less
Taccone et al., 2024 [25]		850	51.5	376 (44.2)	486 (57.2)	7	623/814 (76.5)	84/814 (10.3)	107/814 (13.1)	163.5	8.5	9 g/dLor less	7 g/dLor less
Turgeon et al., 2024^c ^[29]		742	48.7	201 (27.0)	742 (100)	NA	545/724 (75.3)	83/724 (11.5)	96/724 (13.3)	165.6	9.1	10.0 g/dLor less	7.0 g/dLor less
Gobatto et al., 2019 [26]		44	35	4 (9)	44 (100)	4	24 (54)	20 (45)		NA	8	9 g/dLor less	7 g/dLor less
Robertson et al., 2014^b ^[27]		200	29	26 (13)	200 (100)	NA	118 (59)	27 (13.5)	52 (26)	144	14.5	10.0 g/dLor less	7.0 g/dLor less
McIntyre et al., 2006 [28]		67	41	13 (19)	67 (100)	7	NA	NA	NA	NA	NA	10.0 g/dLor less	7.0 g/dLor less

Pooled Analyses of All Included Studies and Sensitivity Analyses

**Figure 2:**
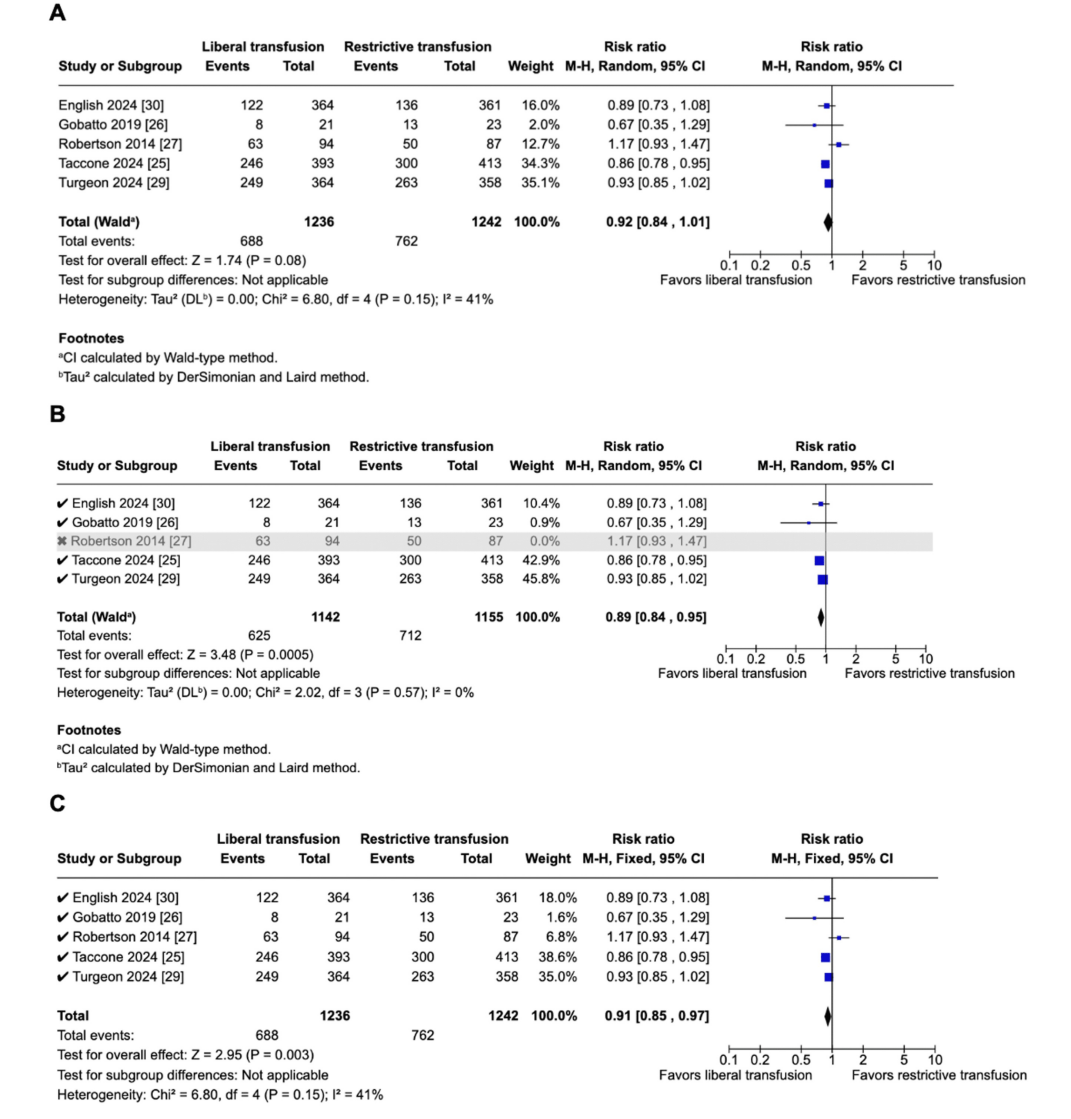
Pooled analysis of unfavorable neurological outcomes A. There was a non-significant decrease in unfavorable neurological outcomes in the liberal group using the random-effects model. B. There was a significant decrease in unfavorable neurological outcomes in the liberal group when leaving out the study by Robertson et al. (2014). C. There was a significant decrease in unfavorable neurological outcomes in the liberal group using the fixed-effects model.

In those allotted to the liberal transfusion strategy, the overall trend was a reduction in unfavorable neurological outcomes (five studies; 1,236 vs. 1,242 patients; 55.7% vs. 61.4%; RR 0.92; 95% CI 0.84-1.01; p = 0.08; I^2^ = 41%; Figure 2A) and shorter hospital stay (three studies; 456 vs. 469 patients; MD -2.43 days; 95% CI, -6.73-1.87; p = 0.27; I2 = 0%), although these differences were not statistically significant. 

Due to high statistical heterogeneity, we performed a leave-one-out analysis by iteratively removing one study at a time to ensure that the results were not dependent on a single study, obtaining statistical significance in unfavorable neurologic endpoints after removing Robertson et al.'s paper (2014) (four studies; 1,142 vs. 1,155 patients; 54.7% vs. 61.6% RR 0.89; 95% CI 0.84-0.95; p = 0.0005; I^2^ = 0%; Figure 2B) [27].

Overall, removing one study at a time did not affect the pooled analysis of the endpoints, ICU mortality, ARDS, and infection rates. There was no statistical heterogeneity in VTE (I² from 52% to 0%) when leaving out the study by Robertson et al. (2014) [27]. We also analyzed the unfavorable neurologic endpoints using the fixed effects model, and we obtained statistical significance in favor of the liberal group (five studies; 1,236 vs. 1,242 patients; 55.7% vs. 61.4%; RR, 0.91; 95% CI 0.85-0.97; p = 0.003; I2 = 41%; Figure 2C).

**Figure 3:**
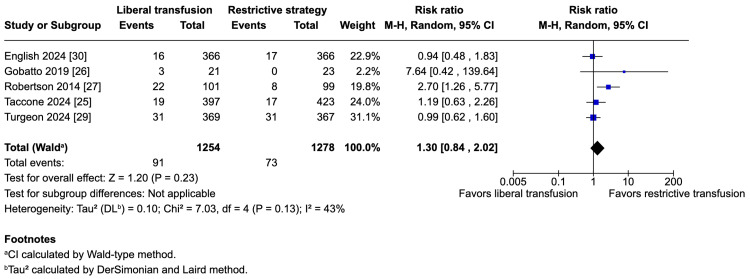
Pooled analysis of venous thromboembolism (VTE)

**Figure 4:**
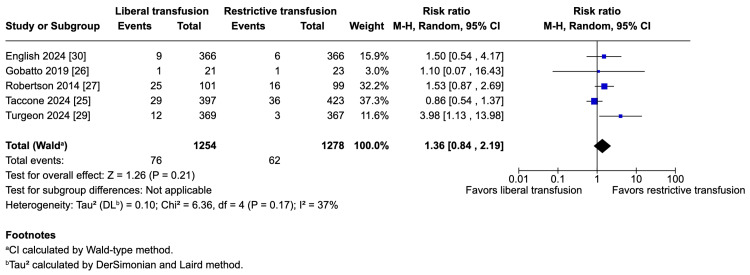
Pooled analysis of acute respiratory distress syndrome (ARDS)

The potential harms of the liberal strategy were an increase in VTE (five studies; 1,254 vs. 1,278 patients; 7.3% vs. 5.7%; RR 1.30; 95% CI 0.84-2.04; p = 0.23; I^2^ = 43%; Figure 3) and an increase in ARDS (five studies; 1,254 vs. 1,278 patients; 6.1% vs. 4.9%; RR 1.36; 95% CI 0.84-2.19; p = 0.21; I^2^ = 37%; Figure 4); however, these results do not strongly favor either strategy because of a lack of statistical significance.

There was no change across both groups in the liberal group for all-cause mortality (six studies; 1,286 vs. 1,290 patients; 25.7% vs. 27.3%; RR 0.95; 95% CI 0.83-1.07; p = 0.39; I^2^ = 0%), ICU mortality (three studies; 428 vs. 419 patients; 15.7% vs. 15.8%; RR 0.74; 95% CI 0.28-1.91; p = 0.53; I^2^ = 48%), and infection rates (six studies; 1,292 vs. 1,307 patients; 39.6% vs. 39.6%; RR 1.00; 95% CI 0.90-1.12; p = 1.00; I^2^ = 18%).

Quality Assessment

**Figure 5:**
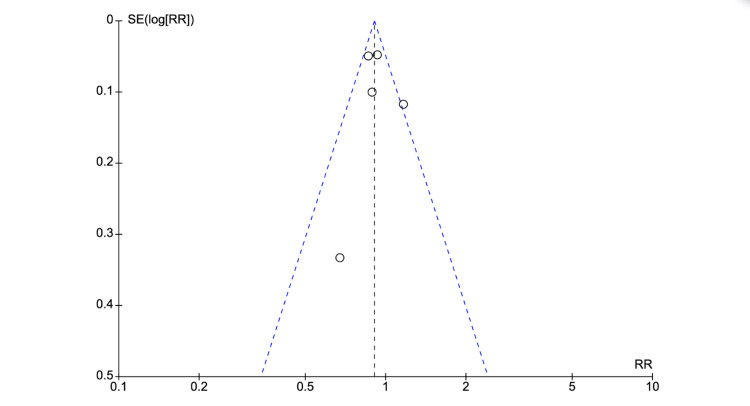
Funnel plot analysis SE: standard error; RR: risk ratios

The ROB 2 tool was used for the quality assessment [15]. No studies were considered to be at high risk for bias. In the funnel plot analysis, studies occupied a symmetrical distribution according to weight and converged towards the pooled effect as the weight increased for unfavorable neurological outcomes (Figure 5). However, there were not enough studies under this analysis to draw any substantial conclusions from the funnel plot analysis regarding publication bias (Figure 5). Thus, publication bias cannot be ruled out [32].

Grade assessment was conducted using the GRADEPro GDT software and reported in Table 2 [21-24].

**Table 2:** GRADE assessment Explanations: ^a^A funnel plot reveals asymmetrical distribution of studies when adjusted by weight; ^b^Confidence interval crosses the null value GRADE: Grading of Recommendations, Assessment, Development, and Evaluation RR: risk ratios Included studies: [25-30]

Outcomes	Anticipated absolute effects^*^ (95% CI)		Relative effect (95% CI)	№ of participants (studies)	Certainty of the evidence (GRADE)
	Risk with restrictive transfusion	Risk with liberal transfusion			
All-cause mortality	273 per 1000	259 per 1000 (226 to 292)	RR 0.95 (0.83 to 1.07)	2576 (6 studies)	⨁⨁⨁◯ Moderate^a^
Any infections	396 per 1000	396 per 1000 (356 to 443)	RR 1.00 (0.90 to 1.12)	2599 (6 studies)	⨁⨁⨁◯ Moderate^b^
Venous thromboembolic events	57 per 1000	74 per 1000 (48 to 115)	RR 1.30 (0.84 to 2.02)	2532 (5 studies)	⨁⨁⨁◯ Moderate^b^
Acute respiratory distress syndrome	49 per 1000	66 per 1000 (41 to 106)	RR 1.36 (0.84 to 2.19)	2532 (5 studies)	⨁⨁⨁◯ Moderate^b^
Unfavorable neurological outcome	614 per 1000	564 per 1000 (515 to 620)	RR 0.92 (0.84 to 1.01)	2478 (5 studies)	⨁⨁⨁◯ Moderate^b^
Length of hospital stay	-	MD 2.43 lower (6.73 lower to 1.87 higher)	-	925 (3 studies)	⨁⨁⨁◯ Moderate^b^
ICU mortality	158 per 1000	117 per 1000 (44 to 301)	RR 0.74 (0.28 to 1.91)	847 (3 studies)	⨁⨁⨁◯ Moderate^b^

Discussion

In this systematic review and meta-analysis of six RCTs, including 2,645 patients, we compared a liberal transfusion strategy with a restrictive transfusion strategy in patients with acute brain injury. The main findings in the liberal transfusion group were (1) a lower risk of unfavorable neurological outcomes, (2) no changes in all-cause mortality and ICU mortality, and (3) an increase in VTE. However, none of these findings were statistically significant in the initial analysis. Post-hoc power calculations determined that our study had an 82% power to detect a 20% difference in unfavorable neurological outcomes.

Nevertheless, there was a significant reduction in unfavorable neurological outcomes at six months in the liberal group in both sensitivity analyses. First, when leaving out a single paper [27], the statistical heterogeneity shifted from I² = 41% to 0%. This suggests that this single paper carried all the statistical heterogeneity observed and thus is potentially subject to bias. We also carried out a Graphic Display of Statistical Heterogeneity (GOSH) analysis and Baujat plot [33], which both pointed to Robertson et al.'s 2014 study being responsible for all statistical heterogeneity observed. The existence of statistical heterogeneity suggests that there may not be a single intervention effect but a variety of intervention effects [34]. A review of the methods in this trial reveals concurrent erythropoietin administration to the patients in addition to the transfusion strategies. This served as a potential confounder and may explain the observed statistical heterogeneity. Moreover, the erythropoietin regimen was switched from a high dose to a lower dose owing to potential safety concerns raised by the US Food and Drug Administration (FDA) based on an erythropoietin multicenter stroke study. In that study, patients who received a dosage regimen similar to that had a higher mortality rate than patients who received a placebo (16.4% vs. 9.0%, p = 0.01) [35]. This also serves as a potential explanation for the high statistical heterogeneity observed in VTE, I² = 43%, which reduced to 0% in a sensitivity analysis, leaving out this paper [27].

Again, fixed-effect meta-analyses ignore statistical heterogeneity. The summary effect estimate from a fixed-effect meta-analysis is normally interpreted as being the best estimate of the intervention effect [34]. Our sensitivity analyses should be interpreted as hypothesis-generating rather than definitive, particularly given the post hoc nature of identifying the study by Robertson et al. (2014) [27] as an outlier. However, the biological plausibility of erythropoietin as an effect modifier lends credence to this approach.

Previous meta-analyses conducted on this topic were unable to detect significant differences between the two strategies, partially due to a lack of high-quality randomized data [10-12]. However, common practice has been geared towards a restrictive approach, as it was previously demonstrated that it reduces the number of units transfused per patient [36].

The findings of this meta-analysis align, in light of current literature, with results from a recent trial of 742 patients with moderate or severe TBI and anemia. A liberal versus restrictive transfusion strategy (transfusion hemoglobin threshold of 10 g/dL versus 7 g/dL) resulted in a non-significant decrease in unfavorable neurologic outcomes at six months (68% versus 73%) but higher rates of ARDS (3.3% versus 0.8%). The mortality and VTE rates were similar [29]. In another trial of 820 patients with TBI or intracerebral or subarachnoid hemorrhage, among the subset with moderate or severe TBI, unfavorable neurologic outcomes at six months were less frequent with a transfusion hemoglobin threshold of 9 g/dL versus 7 g/dL (59% to 67%), as were cerebral ischemic events (9% versus 14%). Mortality and other adverse events were similar between the groups [25]. The most recently published trial focused solely on patients with aneurysmal subarachnoid hemorrhage with anemia; this trial had a longer follow-up time of 12 months as compared to other included studies and did not show significant differences between the two groups [30]. Research is ongoing around this topic, assessing the effects of bundled interventions, including red-cell transfusion, guided by invasive monitoring of brain-tissue oxygenation to improve clinical outcomes [37]. Our analysis revealed clinically important, though statistically nonsignificant, increases in both VTE (RR 1.30) and ARDS (RR 1.36) with liberal transfusion strategies. These findings must be interpreted in the context of the limited statistical power of our meta-analysis. Post-hoc calculations indicate we had only 35% power to detect a 20% relative difference in VTE and 27% power for ARDS at alpha 0.05. This underscores that the apparent non-significance of these safety outcomes may reflect type II error rather than true equivalence. The consistent direction of effect across most included studies (most trials showed increased point estimates for both outcomes with liberal transfusion) suggests these represent genuine safety signals. Particularly concerning is the magnitude of absolute risk increase (17 additional VTE events and ARDS cases per 1,000 patients), which may be clinically meaningful given the catastrophic consequences of these complications in brain-injured patients. These findings align with known pathophysiological mechanisms whereby increased transfusion volume may exacerbate systemic inflammation (ARDS risk) and hypercoagulability (VTE risk). While the current evidence remains inconclusive due to power limitations, the consistency and biological plausibility of these safety signals warrant caution in adopting liberal transfusion thresholds, particularly in high-risk patients. Future trials should prioritize adequate powering for these critical safety outcomes.

Our study has several important limitations. First, the included studies varied in the severity of acute brain injury and other patient characteristics, which may have contributed to the observed statistical heterogeneity in the outcomes. Second, the definitions of ‘liberal’ and 'restrictive’ transfusion strategies varied between studies, potentially limiting the ability to compare the strategies directly. Third, some of the included studies may have been subject to the risk of bias due to deviations from the planned interventions. To mitigate this risk, we conducted a quality assessment using the ROB 2 tool and found all studies to have some concerns for bias based on deviations from protocol. 

Also, some deviations from protocol occurred. We could not carry out the initially intended subgroup analyses as stated in our protocol due to studies not reporting data specific to these population subsets. We initially planned to analyze hazard ratios to preserve time-to-event data, but all included studies reported exclusively risk ratios. The inclusion criteria for the restrictive threshold were changed from 7 g/dL to 8 g/dL to include a recently published large RCT. This decision was taken in the face of the limited number of studies exploring this domain and the need for greater statistical power. Nonetheless, the number of protocol violations was limited. Our expansion of the restrictive threshold criterion, while justified to include important recent evidence, represents a protocol deviation that readers should consider when interpreting results. The included trials employed heterogeneous transfusion thresholds (liberal: 9-10 g/dL; restrictive: 7-8 g/dL), which may affect the generalizability of our findings. While this variability reflects real-world clinical practice, it introduces important considerations. The consistent direction of effect across thresholds implies that clinicians should weigh the same fundamental trade-offs (neurological benefit versus systemic risks) regardless of local protocols. However, institutions using more conservative thresholds (e.g., 8 g/dL restrictive) may observe attenuated effects. The threshold variability precludes definitive recommendations for specific hemoglobin triggers. Instead, our data support individualized decisions based on (1) ischemic risk (e.g., elevated intracranial pressure (ICP), poor collateral circulation), (2) thrombotic/respiratory risk factors (e.g., trauma, prolonged immobility), and (3) monitoring availability (e.g., brain tissue oxygenation probes). Large trials using standardized thresholds (e.g., 7 vs. 9 g/dL) are needed to clarify dose-dependent effects. Pending such data, clinicians should interpret our results in the context of their institutional protocols. While threshold variability complicates universal recommendations, the consistent direction of effects across studies reinforces the need for cautious, patient-centered transfusion strategies.

The inclusion of patients with different types of brain injury raises the possibility that there may be varied susceptibility to cerebral ischemia from anemia. The definition of acute brain injury in this meta-analysis was chosen based on the following points: (1) These are common types of acute brain injuries that often require intensive care management; (2) previous observational studies have shown that hemoglobin levels below 9 g/dL were associated with poorer outcomes in patients with TBI or subarachnoid hemorrhage; and (3) by including these three types of brain injuries, the study could assess the impact of transfusion strategies across a range of acute brain injury conditions, potentially making the results more generalizable.

Moreover, several studies in our analysis had small sample sizes, which may have increased the risk of overestimating the treatment effects or contributing to publication bias. The risk of publication bias was analyzed using a funnel plot. The use of a random-effects model, while appropriate for accounting for statistical heterogeneity, may have overestimated the treatment effect because of the influence of small studies with large variances. We addressed this issue by performing a sensitivity analysis using a fixed effects model. 

The follow-up periods in the included studies were relatively short, and long-term outcomes, such as cognitive function, quality of life, and disability, were not consistently assessed, limiting the ability to conclude the long-term effects of transfusion strategies. The studies included in our meta-analysis primarily focused on adult patients with acute brain injury, limiting the applicability of our findings to pediatric populations or those with significant comorbidities. 

## Conclusions

This meta-analysis of 2,645 patients suggests that there is no substantial difference in outcomes between a liberal and a restrictive transfusion strategy in patients with acute brain injury. In light of these findings, we suggest that while a liberal strategy may offer neurological benefits for some patients, the current evidence remains insufficient to recommend widespread adoption. Clinicians should consider individual patient factors and institutional resources when making transfusion decisions. Large, pragmatic RCTs with standardized thresholds are needed to resolve this clinical equipoise.

## Appendices

Appendix A

Full Search String

PubMed: ("Traumatic Brain injury" OR "Acute brain injury" OR "Head trauma" OR "Brain Injuries"[MeSH] OR "Subarachnoid Hemorrhage" OR "Subarachnoid Hemorrhage"[MeSH]) AND (Liberal OR Higher) AND ("Blood Transfusion" OR Hemoglobin OR "Blood Transfusion"[MeSH] OR "Blood products" OR "Packed Red Blood Cells")

Embase: ("Traumatic Brain injury" OR "Acute brain injury" OR "Head trauma" OR "Brain Injuries" OR "Subarachnoid Hemorrhage") AND (Liberal OR Higher) AND ("Blood Transfusion" OR Hemoglobin OR "Blood products" OR "Packed Red Blood Cells")

Cochrane Library: ("Traumatic Brain injury" OR "Acute brain injury" OR "Head trauma" OR "Brain Injuries" OR "Subarachnoid Hemorrhage") AND (Liberal OR Higher) AND ("Blood Transfusion" OR Hemoglobin OR "Blood products" OR "Packed Red Blood Cells")

Appendix B

Reasons for Exclusion After Full-Text Review

Wrong population: Kosaki Y, Hongo T, Hayakawa M, Kudo D, Kushimoto S, Tagami T, Naito H, Nakao A, Yumoto T. Association of initial lactate levels and red blood cell transfusion strategy with outcomes after severe trauma: a post hoc analysis of the RESTRIC trial. World J Emerg Surg. 2024 Jan 2;19(1):1. doi: 10.1186/s13017-023-00530-7. PMID: 38167057; PMCID: PMC10763143; Hayakawa M, Tagami T, Kudo D, et al. The Restrictive Red Blood Cell Transfusion Strategy for Critically Injured Patients (RESTRIC) trial: a cluster-randomized, crossover, non-inferiority multicenter trial of restrictive transfusion in trauma. J Intensive Care. 2023;11(1):34. doi:10.1186/s40560-023-00682-3

Wrong intervention: Naidech AM, Shaibani A, Garg RK, Duran IM, Liebling SM, Bassin SL, Bendok BR, Bernstein RA, Batjer HH, Alberts MJ. Prospective, randomized trial of higher goal hemoglobin after subarachnoid hemorrhage. Neurocrit Care. 2010 Dec;13(3):313-20. doi: 10.1007/s12028-010-9424-4. PMID: 20717750; Zygun DA, Nortje J, Hutchinson PJ, Timofeev I, Menon DK, Gupta AK. The effect of red blood cell transfusion on cerebral oxygenation and metabolism after severe traumatic brain injury. Crit Care Med. 2009 Mar;37(3):1074-8. doi: 10.1097/CCM.0b013e318194ad22. PMID: 19237920.

Full text unavailable: Audibert, G.1; Charpentier, C.1; Crumière, P.-P.1; Cantais, E.2; Joly, L.-M.3; Mertes, P.-M.4. Effect of transfusion on cerebral metabolism after severe traumatic brain injury: liberal versus restrictive strategy: 7AP4-4. European Journal of Anaesthesiology 31():p 118, June 2014; ChiCTR2400083713. Effect of different red blood cells transfusion strategies on the outcome of neurocritical patients: a randomized controlled study. https://trialsearch.who.int/Trial2.aspx?TrialID=ChiCTR2400083713. Published online 2024. https://www.cochranelibrary.com/central/doi/10.1002/central/CN-02696965/full

Protocol: Taccone FS, Badenes R, Rynkowski CB, Bouzat P, Caricato A, Kurtz P, Moller K, Diaz MQ, Van Der Jagt M, Videtta W, Vincent JL. TRansfusion strategies in Acute brain INjured patients (TRAIN): a prospective multicenter randomized interventional trial protocol. Trials. 2023 Jan 7;24(1):20. doi: 10.1186/s13063-022-07061-7. Erratum in: Trials. 2024 Jun 23;25(1):410. doi: 10.1186/s13063-024-08282-8. PMID: 36611210; PMCID: PMC9825124; Turgeon AF, Fergusson DA, Clayton L, Patton MP, Zarychanski R, English S, Docherty A, Walsh T, Griesdale D, Kramer AH, Scales D, Burns KEA, Boyd JG, Marshall JC, Kutsogiannis DJ, Ball I, Hébert PC, Lamontagne F, Costerousse O, St-Onge M, Lessard Bonaventure P, Moore L, Neveu X, Rigamonti A, Khwaja K, Green RS, Laroche V, Fox-Robichaud A, Lauzier F; HEMOTION Trial Team, the Canadian Critical Care Trials Group, the Canadian Perioperative Anesthesia Clinical Trials Group and the Canadian Traumatic Brain Injury Research Consortium. Haemoglobin transfusion threshold in traumatic brain injury optimisation (HEMOTION): a multicentre, randomised, clinical trial protocol. BMJ Open. 2022 Oct 10;12(10):e067117. doi: 10.1136/bmjopen-2022-067117. PMID: 36216432; PMCID: PMC9557781.

Overlapping populations: Vedantam A, Yamal JM, Rubin ML, Robertson CS, Gopinath SP. Progressive hemorrhagic injury after severe traumatic brain injury: Effect of hemoglobin transfusion thresholds. J Neurosurg. 2016 Nov;125(5):1229-1234. doi: 10.3171/2015.11.JNS151515. Epub 2016 Mar 4. PMID: 26943843; PMCID: PMC5065393; Yamal JM, Rubin ML, Benoit JS, Tilley BC, Gopinath S, Hannay HJ, Doshi P, Aisiku IP, Robertson CS. Effect of Hemoglobin Transfusion Threshold on Cerebral Hemodynamics and Oxygenation. J Neurotrauma. 2015 Aug 15;32(16):1239-45. doi: 10.1089/neu.2014.3752. Epub 2015 Mar 26. PMID: 25566694; PMCID: PMC4532899; Gopinath SP, Hannay J, Yamal JM, Goodman JC, Tilley B, Robertson C. Thromboembolic complications associated with a transfusion threshold of 10 g/dl in tbi patients. J Neurosurg. 2015;122(6):A1557. doi:10.3171/2015.6.JNS.AANS2014abstracts

Observational study: Kurtz P, Taccone F, Gonçalves B, et al. Predictors of mortality after subarachnoid hemorrhage: A retrospective multicenter cohort study. Crit Care. 2019;23((Kurtz P.; Gonçalves B.; Shinotsuka C.) IECPN-Instituto Estadual do Cérebro Paulo Niemeyer, Rio da Janeiro, RJ, Brazil). doi:10.1186/s13054-019-2503-9

Conference abstracts: Kramer A, Diringer M, Naidech A, Macdonald L, LeRoux P. Hemoglobin thresholds for a clinical trial comparing liberal and restrictive transfusion strategies in patients with aneurysmal subarachnoid hemorrhage: a multidisciplinary North American survey. Neurocrit Care. 2010;13:S207. doi:10.1007/s12028-010-9426-2

**Table 3:** Risk of bias in the included studies. All studies were estimated to have some concern for bias due to unjustified deviations from protocol in transfusion thresholds owing to trial design where physicians had to be unblind.

Study	Bias from randomization process	Bias due to deviations from intended interventions	Bias due to missing outcome data	Bias in measurement of the outcomes	Bias in selection of the reported result	Overall risk of bias
English et al., 2024 [30]	LOW	SOME CONCERN	LOW	LOW	LOW	SOME CONCERN
Taccone et al., 2024 [25]	LOW	SOME CONCERN	LOW	LOW	LOW	SOME CONCERN
Turgeon et al., 2024 [29]	LOW	SOME CONCERN	LOW	LOW	LOW	SOME CONCERN
Gobatto et al., 2019 [26]	LOW	SOME CONCERN	LOW	LOW	LOW	SOME CONCERN
Robertson et al., 2014 [27]	LOW	SOME CONCERN	LOW	LOW	LOW	SOME CONCERN
McIntyre et al., 2006 [28]	LOW	SOME CONCERN	LOW	LOW	LOW	SOME CONCERN
